# Supplementary material for: Exploring Factors Associated with Health Status and Dietary Supplement Use Among Portuguese Adults: A Cross-Sectional Online Survey
Source: Healthcare (Basel). 2025 Mar 30;13(7):769. doi: 10.3390/healthcare13070769 (PMC11988924; doi:10.3390/healthcare13070769)
Supplement: Supplementary file 1 [file healthcare-13-00769-s001.zip › healthcare-3529330-supplementary.pdf]

SUPPLEMENTARY MATERIAL

Table S1. Sociodemographic characteristics of participants and frequencies of responses on health status and self-care practices (medication use, dietary supplementation and herbal infusion consumption)

Variables	Variable code	Total, N (%)
Sex		449 (100)
Female		330 (73)
Male		119 (27)
Age groups (years)		449 (100)
18-29		176 (39)
30-39		86 (19)
40-60		154 (34)
60+		33 (7)
Education level		449 (100)
Basic		8 (2)
Secondary		64 (14)
Post-secondary		17 (4)
Higher		360 (80)
Diagnosed disease		449 (100)
No		288 (64)
Yes		161 (36)
One diagnosed disease, type*		95 (100)
Gastrointestinal disorders		15 (16)
Heart diseases		9 (9)
Dyslipidemia		8 (8)
Hypertension		6 (6)
Cancer		4 (4)
Kidney diseases		3 (3)
Liver diseases		2 (2)
Other		48 (51)
Medication use		449 (100)
No		335 (75)
Yes		114 (25)
Medication, N		
1 medication	1	84 (74)
2 medications	2	20 (18)
≥ 3 medications	3	10 (9)

Cardiovascular drug, type	CV drug, N	114
No use	no use	89 (78)
Antihypertensive	1	16 (14)
Antidyslipidemic		4 (4)
Antihypensive + antidyslipidemic	2	5 (4)
Blood drug, type		114
No use		102 (89)
Anticoagulant		12 (11)
Endocrine drug, type	Endocrine drug, N	114
No use	no use	49 (43)
Oral contraceptive	1	55 (48)
Thyroid hormone		5 (4)
Antidiabetic		2 (2)
Oral contraceptive + thyroid hormone	2	2 (2)
Oral contraceptive + antiandrogenic		1 (1)
Dietary supplements use		449
No		220 (49)
Yes		229 (51)
Dietary supplements, type		229
Multivitamin-mineral supplements	1	132 (29)
Other dietary supplements	2	44 (10)
Both supplement types	3	53 (12)
Herbal infusions		449
No		34 (8)
Yes		415 (92)

* Excluding mental health conditions

Table S2. Results of the Chi-square teste for dietary supplements use and herbal infusion consumption by sex.

		95% Confidence Intervals		
Sex	Odds ratio (OR)	Lower	Upper	*p* value
Female	1.28	0.48	3.42	0.23
Male	1.00	0.32	3.09	

Table S3. Health status and self-care practices of participants, stratified by age groups: Chi-square *p* values and Cramér V values presented

		Age group (years), N (%)					
	Total, N = 449	18-29	30-39	40-60	+ 60	*p* value	Cramér's V
Diagnosed disease						< 0.001	0.22
No	288 (64)	129 (73)	58 (67)	90 (58)	11 (33)		
Yes	161 (36)	47 (27)	28 (33)	64 (42)	22 (67)		
Diagnosed disease, N						< 0.001	0.33
1 disease	118 (73)	41 (87)	22 (79)	47 (73)	8 (36)		
2 diseases	22 (14)	6 (13)	4 (14)	9 (14)	3 (14)		
≥ 3 diseases	21 (13)	0	2 (1)	8 (13)	11 (50)		
Mental health conditions		13 (28)	8 (29)	17 (27)	8 (36)	0.84	--
BMI categories						0.14	--
Normal	286 (63)	119 (68)	60 (70)	88 (57)	17 (52)		
Overweight	110 (25)	37 (21)	18 (21)	45 (29)	10 (30)		
Obese	44 (10)	13 (7)	6 (7)	19 (12)	6 (18)		
Underweight	10 (2)	6 (3)	2 (2)	2 (1)	0		
Self-perceived health						< 0.001	0.21
Good	310 (69)	121 (69)	64 (74)	107 (69)	18 (55)		
Very good	83 (18)	44 (25)	17 (20)	19 (12)	3 (9)		
Fair	57 (12)	11 (6)	5 (6)	28 (18)	12 (36)		
Medication use	114 (25)	42 (24)	20 (23)	34 (22)	18 (55)	0.001	0.19
Medication, N						0.33	--
1 medication	84 (74)	34 (81)	16 (80)	25 (74)	9 (50)		
2 medications	20 (18)	5 (12)	3 (15)	6 (18)	6 (33)		
≥ 3 medications	10 (9)	3 (7)	1 (5)	3 (9)	3 (17)		
Dietary supplements use							
Yes	229 (51)	76 (43)	49 (57)	82 (53)	22 (67)	0.028	0.14
Dietary supplement, type						0.066	--

(1) Multivitamin-mineral supplements	132 (29)	42 (24)	30 (35)	44 (29)	16 (48)
(2) Other supplements	44 (10)	19 (11)	8 (9)	6 (10)	1 (3)
(3) Both type supplemments	53 (12)	15 (9)	11 (13)	22 (14)	5 (15)

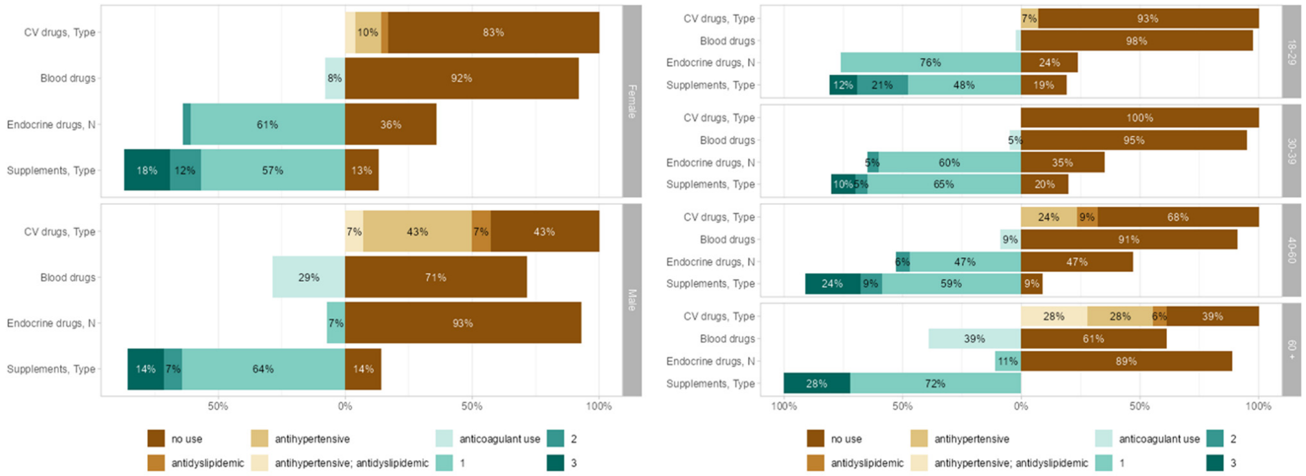

Figure S1. Distribution of therapeutic medication categories by sex and age group. (A) By sex. (B) By age group. Cardiovascular (CV).

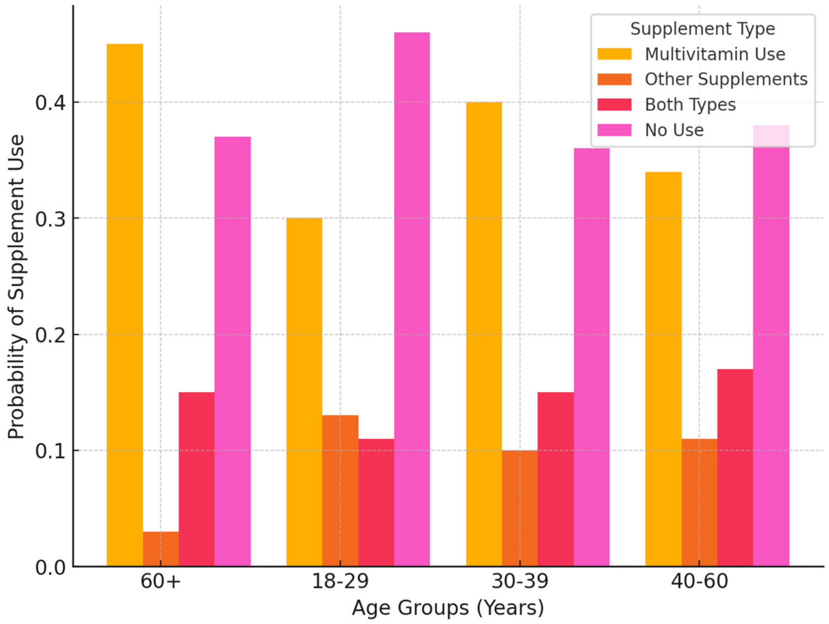

Figure S2. Bar chart displaying the estimated marginal means (EMMs) of dietary supplement use across different age groups. Each bar represents the probability of using a specific supplement type, with age groups on the x-axis. The 60+ age group have the highest probability

of multivitamin-mineral supplement use, while participants in the 18-29 age group exhibit the highest probability of non-use.

Table S4. Partial correlation analysis between self-perceived health and other variables

		Self-perceived health	Diagnosed disease, N	BMI	Medication, N
Self-perceived health	Pearson's r	—	—	—	—
	p-value	—	—	—	—
Diagnosed disease, N	Pearson's r	-0.28**	—	—	—
	p-value	< 0.001	—	—	—
BMI	Pearson's r	-0.20*	0.06	—	—
	p-value	0.013	0.418	—	—
Medication, N	Pearson's r	-0.08	0.10	0.11	—
	p-value	0.527	0.418	0.344	—

Note. controlling for “Mental health conditions”. * $p < 0.05$, ** $p < 0.001$

Table S5. Partial correlation analysis between the number of medications use and the type of medication use by participants

		Medication, N	Digestive system drugs, N	Cardiovascular drugs, N	Endocrine drugs, N
Medication, N	Pearson's r	—	—	—	—
	p-value	—	—	—	—
Digestive system drugs, N	Pearson's r	0.17	—	—	—
	p-value	0.08	—	—	—
Cardiovascular drugs, N	Pearson's r	0.40**	-0.08	—	—
	p-value	< 0.001	0.39	—	—

Endocrine drugs, N	Pearson's r	-0.07	-0.18	-0.43**	—
	p-value	0.44	0.06	< 0.001	—

Note. ** *p* < 0.001. N, number

Table S6. Results of the multinomial logistic regression regarding the association of age, **sex**, number of diagnosed diseases, BMI categories and medication use with self-perceived health, whereas “good” is the reference category

	Very good		Fair	
	Unadjusted	Adjusted	Unadjusted	Adjusted
	OR (95%CI)	OR (95%CI)	OR (95%CI)	OR (95%CI)
**Sex**				
Male (vs Female)	0.94 (0.54, 1.64)	0.0 (0.0, 0.0)	1.11 (0.59, 2.09)	1.40 (0.24, 8.24)
Age groups, years				
18-29 (vs 60+)	2.18 (0.61, 7.77)	0.08 (0.0, 2.12)	0.14 (0.05, 0.35)	1.43 (0.14, 14.56)
30-39 (vs 60+)	1.59 (0.42, 6.05)	0.81 (0.04, 14.74)	0.12 (0.04, 0.38)	0.20 (0.01, 3.37)
40-60 (vs 60+)	1.07 (0.29, 3.97)	0.10 (0.0, 2.26)	0.39 (0.17, 0.91)	1.04 (0.16, 6.78)
Diagnosed disease, N				
2 (vs 1 disease)	1.29 (0.25, 6.49)	1.50 (0.13, 17.36)	2.14 (0.73, 6.32)	17.26 (1.59, 187.30)
≥ 3 (vs 1 disease)	0	0	5.50 (2.04, 14.88)	24.99 (2.42, 257.80)
BMI, categories				
Overweight (vs normal)	0.68 (0.36, 1.27)	3.22 (0.36, 20.10)	2.48 (1.30, 4.74)	17.18 (2.66, 110.89)
Obese (vs normal)	0.62 (0.23, 1.68)	NaN	3.88 (1.74, 8.69)	61.48 (3.41, 1109)
Medication, N				
2 (vs 1 medication)	0.81 (0.20, 3.24	1.45 (0.15, 14.40)	0.72 (0.21, 2.46)	0.12 (0.02, 0.86)
≥ 3 (vs 1 medication)	1.17 (0.11, 12.11)	1.03 (0.05, 20.64)	4.67 (1.07, 20.44)	24.99 (2.42, 257.8)
